# Lack of Desmin in Mice Causes Structural and Functional Disorders of Neuromuscular Junctions

**DOI:** 10.3389/fnmol.2020.567084

**Published:** 2020-10-26

**Authors:** Nane Eiber, Franziska Fröb, Mirjam Schowalter, Christian Thiel, Christoph S. Clemen, Rolf Schröder, Said Hashemolhosseini

**Affiliations:** ^1^Institute of Biochemistry, Friedrich-Alexander-University of Erlangen-Nürnberg, Erlangen, Germany; ^2^Institute of Neuropathology, University Hospital Erlangen, Friedrich-Alexander-University of Erlangen-Nürnberg, Erlangen, Germany; ^3^Medical Faculty, Institute of Human Genetics, Friedrich-Alexander-University of Erlangen-Nürnberg, Erlangen, Germany; ^4^Muscle Research Center Erlangen (MURCE), Friedrich-Alexander-University of Erlangen-Nürnberg, Erlangen, Germany; ^5^Institute of Aerospace Medicine, German Aerospace Center (DLR), Cologne, Germany; ^6^Medical Faculty, Center for Physiology and Pathophysiology, Institute of Vegetative Physiology, University of Cologne, Cologne, Germany

**Keywords:** desminopathy, skeletal muscle, neuromuscular junction, desmin, nicotinic acetylcholine receptor, postsynaptic gene, myasthenic syndrome

## Abstract

Desmin, the major intermediate filament (IF) protein in muscle cells, interlinks neighboring myofibrils and connects the whole myofibrillar apparatus to myonuclei, mitochondria, and the sarcolemma. However, desmin is also known to be enriched at postsynaptic membranes of neuromuscular junctions (NMJs). The pivotal role of the desmin IF cytoskeletal network is underscored by the fact that over 120 mutations of the human *DES* gene cause hereditary and sporadic myopathies and cardiomyopathies. A subgroup of human desminopathies comprises autosomal recessive cases resulting in the complete abolition of desmin protein. In these patients, who display a more severe phenotype than the autosomal dominant cases, it has been reported that some individuals also suffer from a myasthenic syndrome in addition to the classical occurrence of myopathy and cardiomyopathy. Since further studies on the NMJ pathology are hampered by the lack of available human striated muscle biopsy specimens, we exploited homozygous desmin knock-out mice which closely mirror the striated muscle pathology of human patients lacking desmin protein. Here, we report on the impact of the lack of desmin on the structure and function of NMJs and the transcription of genes coding for postsynaptic proteins. Desmin knock-out mice display a fragmentation of NMJs in soleus, but not in the extensor digitorum longus muscle. Moreover, soleus muscle fibers show larger NMJs. Further, transcription levels of acetylcholine receptor (AChR) genes are increased in muscles from desmin knock-out mice, especially of the AChRγ subunit, which is known as a marker of muscle fiber regeneration. Electrophysiological recordings depicted a pathological decrement of nerve-dependent endplate potentials and an increased rise time of the nerve-independent miniature endplate potentials. The latter appears related to the fragmentation of NMJs in desmin knockout mice. Our study highlights the essential role of desmin for the structural and functional integrity of mammalian NMJs.

## Introduction

Mutations of the human desmin gene on chromosome 2q35 cause a wide variety of hereditary and sporadic myopathies and cardiomyopathies (Clemen et al., 2013). Desminopathies exist in autosomal-dominant and -recessive subforms. While the latter usually display a childhood-onset and a more severe cardiac and skeletal muscle phenotype, the autosomal-dominant forms are typically characterized by an adult-onset between the third and the fourth decade of life (Clemen et al., 2013). Desmin, the major intermediate filament (IF) protein of muscle cells, serves to maintain myofibrillar cytoarchitecture and distributes externally applied mechanical stress intracellularly (Paulin and Li, 2004). Desmin and associated IFs form a 3-dimensional scaffold around Z-disks whilst linking myofibrils and mechanically and functionally connecting them to nuclei, mitochondria, and sarcolemma (Schröder and Schoser, 2009). Over the last two decades, more than 120 disease-causing desmin mutations have been described; with the human R350P desmin mutation being the most frequently reported gene defect in desminopathies (Clemen et al., 2015,^1^).

The recessive desminopathies can further be divided into subgroups. In one group mutant desmin protein is expressed, whereas. in the other, desmin protein expression is completely abolished (Carmignac et al., 2009; Henderson et al., 2013). Desmin knock-out mice (Milner et al., 1996; Li et al., 1997), which were published long before the first report on a human patient lacking desmin are disease models for the second group (Li et al., 1996). Notably, one study already demonstrated that ablation of desmin protein expression in mice leads to a disorganization of the Neuromuscular junctions (NMJs; Agbulut et al., 2001).

Recently, two cousins with severe, infantile-onset and generalized muscle weakness and fatigability were reported in whom a homozygous DES truncating mutation leads to the complete lack of desmin protein (Durmus et al., 2016). In addition to myopathy and cardiomyopathy, these patients developed a myasthenic syndrome, which was partially improved in response to salbutamol treatment. Electrophysiological analyses of the above-mentioned cousins revealed a decremental response over 10% on repetitive nerve stimulation, indicated a clinically relevant neuromuscular NMJ transmission defect (Durmus et al., 2016).

Since appropriate human skeletal muscle biopsies from intercostal muscles are not available from desmin knock-out patients, we studied the structural and functional NMJ pathology in desmin knock-out mice.

## Materials and Methods

### Mice Strains

Homozygous desmin knock-out mice (B6J.129Sv-*Des*^tm1Cba^/Cscl^2^; breeding pairs were received by courtesy from Denise Paulin, Université Pierre et Marie Curie, Paris, France; Li et al., 1996) and wild-type littermates aged 3 to 4, as well as 7 to 8 months of age, were used. Mouse experiments were performed following animal welfare laws and approved by the responsible local committee [animal protection officer, Sachgebiet Tierschutzangelegenheiten, FAU Erlangen-Nürnberg, AZ: I/39/EE006, TS-07/11). Mice were housed in cages that were maintained in a room with temperature 22 ± 1°C and relative humidity 50–60% on a 12 h light/dark cycle. Water and food were provided *ad libitum*.

### Newton Meter

Muscle force was measured with all four limbs by Grip Strength Test Meter (Bioseb, Chaville, France).

### Electrophysiology

#### Nerve-Muscle Preparations and Extracellular Recording

Electrophysiological recordings were essentially done as previously described (Kravic et al., 2016, 2018). Isolated diaphragm-phrenic nerve preparations were maintained in Liley’s solution gassed with 95% O_2_ and 5% CO_2_ at room temperature (Liley, 1956). The recording chamber had a volume of ca. 1 ml and was perfused at a rate of 1 ml/min. The nerve was drawn up into a suction electrode for stimulation with pulses of 0.1 ms duration. The preparation was placed on the stage of a Zeiss Axio Examiner Z1 microscope fitted with incident light fluorescence illumination with filters for red (Zeiss filter set 20) fluorescing fluorophore (Carl Zeiss MicroImaging, Göttingen). At the beginning of the experiment, the compound muscle action potential (cMAP) was recorded using a micropipette with a tip diameter of ca. 10 μm, filled with a bathing solution. The electrode was positioned so that the latency of the major negative peak was minimized. The electrode was then positioned 100 μm above the surface of the muscle and cMAP was recorded.

#### Intracellular Recording and Data Analysis

To block muscle action potentials, so that endplate potentials (EPPs) and endplate currents (EPCs) could be recorded (Plomp et al., 1992; Rogozhin et al., 2008) μ-conotoxin GIIIB (μ-CTX, 2 μM; Peptide Institute, Osaka) was added to Liley’s solution. At the same time, AChRs were labeled by adding 0.5 × 10^−8^ M of rhodamine-α-bungarotoxin (BTX; Life Technologies, Darmstadt) to the same solution. In some experiments, the effect of the toxin wore off after 1–2 h and contractions resumed in response to nerve stimulation. These preparations were then exposed a second time to the toxin. Two intracellular electrodes (resistance 10–15 MΩ) were inserted within 50 μm of the NMJs under visual inspection (Rogozhin et al., 2008). The current was passed through one electrode to maintain the membrane potential within 2 mV of −75 mV while voltage transients were recorded with the other. Signals were amplified by an Axoclamp 900A and digitized at 40 kHz by a Digidata 1440A under the control of pCLAMP 10 (Molecular Devices, Sunny Vale). Voltage records were filtered at 3 kHz and current records at 1 kHz (8-pole Bessel filter). Current transients were recorded using the two-electrode voltage-clamp facility of the Axoclamp 900A. Clamp gains were usually 300–1,000, reducing the voltage transients to <3% of their unclamped amplitudes. At most NMJs, 50–100 spontaneous quantal events were recorded for 1 min. Records were analyzed using pCLAMP 10. Spontaneous events were extracted using the “template search” facility and edited by the eye to remove obvious artifacts. Events recorded from each NMJ were averaged and the amplitude, rise time, and single exponential decay time were determined.

### 3D Imaging and Analysis

3D Imaging was essentially performed as previously described (Durmus et al., 2016; Kravic et al., 2016). Mice were killed by CO_2_ affixation. Mouse soleus and extensor digitorum longus muscles were dissected and fixed in 2% PFA for 2 h at 4°C. Muscle bundles containing 5–10 fibers were prepared and stained with BTX 1:2,500 (Invitrogen) for 1 h at room temperature. Stained bundles were washed three times 5 min in PBS and embedded in Mowiol.

3D images of NMJs were taken with a 40× oil objective (Zeiss Examiner E1). Images were deconvolved and analyzed using different modules in AxioVision Software. The following parameters were determined for each NMJ: volume, surface, gray sum, gray mean, and the number of fragments. More than 100 NMJs were analyzed.

### GO and Gene Set Enrichment Analysis

RNA sequencing was performed on RNA extracted from the gastrocnemius muscle of 3-month-old wild-type and desmin knock-out mice and is available by GEO accession number GSE154573^3^. RNA was extracted from five gastrocnemius muscle per genotype and pooled to reduce variability. Raw data files (fastq) were used to generate Bam files. Bam files of wild-type and desmin knock-out samples were uploaded to the Galaxy Server^4^ and gene expression was measured using the tool “feature counts” (Galaxy Version 1.6.4 + galaxy1) and an annotation file of mouse genes (mm10). Feature counts were further used to determine differentially expressed genes by “DESeq2” (Galaxy Version 2.11.40.5). Significantly regulated genes were ranked according to their log2 fold change. Genes with a log2 fold change “≥1” or “≤−1” were submitted to the Gene Ontology enRIchment anaLysis and visuaLizAtion tool (GOrilla^5^) and subsequently analyzed using REVIGO^6^. For GSEA, the list of ranked genes was submitted to the Gene Set Enrichment Analysis tool (GSEA^7^, Mootha et al., 2003; Subramanian et al., 2005) using a gene-set list of genes specific for the GO term “NMJ” obtained from AMIGO^8^).

### Statistical Analysis

Data are presented as the mean values, and the error bars indicate ± SEM. The significance is calculated by unpaired, two-tailed *t*-test, or as indicated by the figure legends, and provided as real *P*-values that are believed to be categorized for different significance levels, like, ***P < 0.001, **P < 0.01, or *P < 0.05.

## Results

### Desmin Knock-Out Mice Show Reduced Muscle Strength and Have a Lower Body Weight With Advancing Age

**Figure 1:**
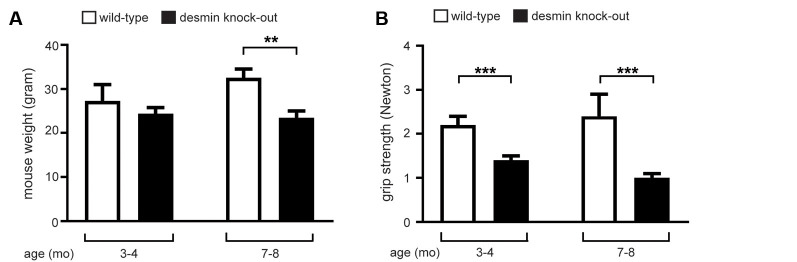
Desmin knock-out mice show reduced muscle strength and have a lower body weight with advancing age. **(A)** For 3–4 months old desmin knock-out mice, a total of three wild-types and four mutant mice were measured. For 7–8 months old desmin knock-out mice, a total of four wild-types and five mutant mice were measured. Statistical significance was determined by unpaired two-tailed *t*-test (**P < 0.01). **(B)** Newton meter measurements of muscle force of desmin mutant mice showed significant muscle weakness. For 3–4 months old desmin knock-out mice, a total of three wild-types and four mutant mice were measured. For 7–8 months old desmin knock-out mice, a total of four wild-types and five mutant mice were measured. Statistical significance was determined by unpaired two-tailed *t*-test (***P < 0.001).

First, we asked for phenotypical changes of desmin knock-out mice in comparison with controls. Based on patient data, we expect a loss of muscle grip strength in the desmin knock-out mice. This might be accompanied by an impact on the bodyweight of the mice. Moreover, since pathological disorders of the skeletal muscles are often observed being age-dependent, two age stages of the mice were examined, i.e., 3–4 and 7–8-month-old mice. The weight of the mice turned out to be reduced at both stages of age (Figure 1). The decrease in body weight in 3–4-month-old mice was indicated, but not statistically significant (Figure 1A). In 7–8-month-old mice, the bodyweight of the mutant mice dropped by more than 25% compared to the controls (Figure 1A). This observed decrease in weight of the desmin knock-out mice correlated with a decrease in muscle strength (Figure 1B). At the age of 3–4 months, a significant decrease of muscle grip strength was measured in desmin knock-out mice in comparison with controls, and this decrease was even more prominent at the age of 7–8 months with approx. 30% (Figure 1B).

### The NMJs of Desmin Knock-Out Mice Show a Higher Degree of Fragmentation

**Figure 2:**
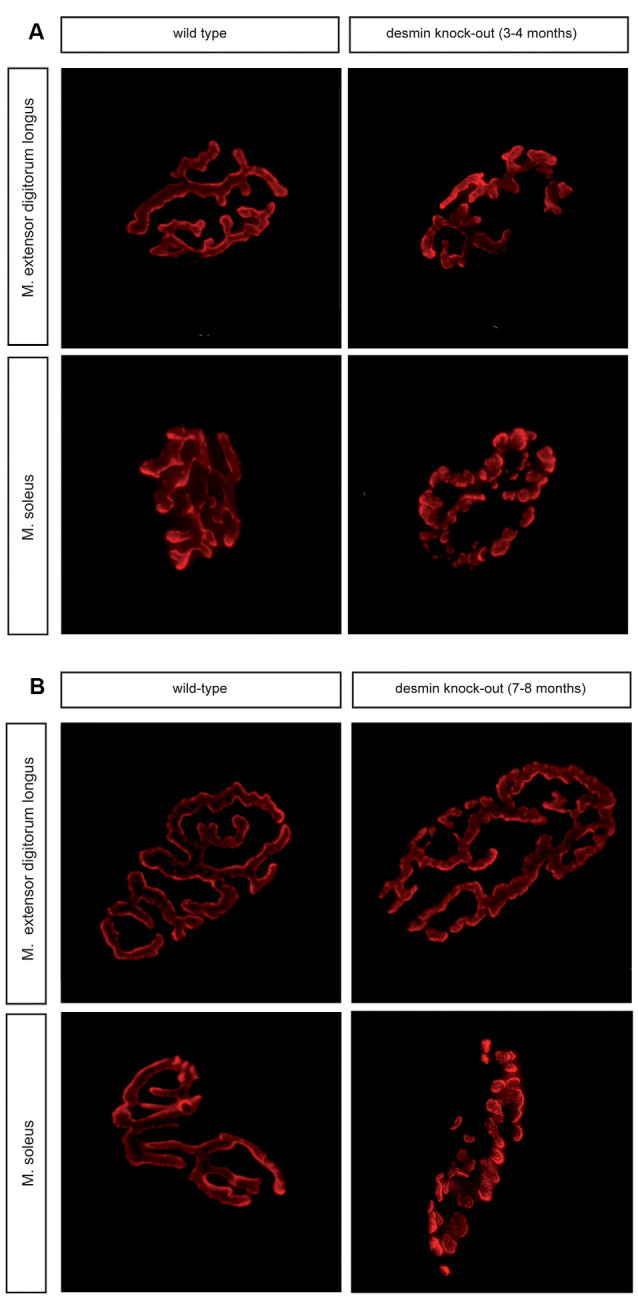
Soleus muscle of desmin knock-out mice show a higher degree of fragmentation of their neuromuscular junctions (NMJs). **(A)** Representative images of BTX wild-type and desmin knock-out homozygous NMJs of oxidative soleus and glycolytic extensor digitorum longus muscles are shown. Note, NMJs of oxidative soleus muscle are fragmented in the absence of desmin. 3–4 months old mice were used. **(B)** Representative images of BTX wild-type and desmin knock-out homozygous NMJs of oxidative soleus and glycolytic extensor digitorum longus muscles are shown. Note, NMJs of oxidative soleus muscle are fragmented in the absence of desmin. 7–8 months old mice were used.

**Figure 3:**
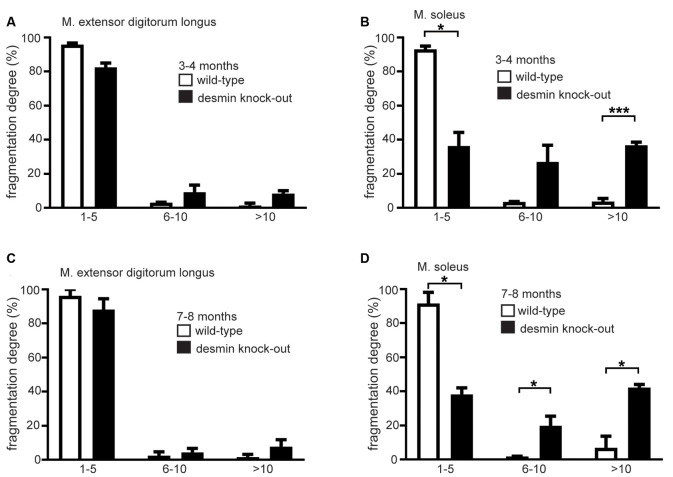
The soleus muscle of desmin knock-out mice shows a higher degree of fragmentation of their NMJs. **(A,B)** The extensor digitorum longus or soleus muscle NMJs were categorized in groups according to their number of fragments. The number of NMJs in each group is given as the percentage of the total NMJ number for each genotype. For extensor digitorum longus a total of 96 wild-types and 89 homozygous, for soleus muscle 90 wild-type and 103 homozygous NMJs were counted (*n* = 3 mouse pairs). Mice were 3–4 months old. Statistical significance was determined by unpaired two-tailed *t*-test (*P < 0.05, ***P < 0.001). **(C,D)** The extensor digitorum longus or soleus muscle NMJs were categorized in groups according to their number of fragments. The number of NMJs in each group is given as the percentage of the total NMJ number for each genotype. For extensor digitorum longus a total of 102 wild-types and 95 homozygous, for soleus muscle 99 wild-type and 102 homozygous NMJs were counted (*n* = 3 mouse pairs). Mice were 7–8 months old. Statistical significance was determined by unpaired two-tailed *t*-test (*P < 0.05).

Many perturbations of NMJs lead to structural disorders and are often represented by fragmentation of NMJs (Li et al., 2018). Here, NMJs of soleus and extensor digitorum longus muscles were analyzed in 3–4 and 7–8 month old desmin knock-out mice. Immunofluorescence stains of muscle fiber bundles with BTX (rhodamine-α-bungarotoxin) revealed NMJs of extensor digitorum longus muscles being non-fragmented in comparison with controls regardless of the age (Figures 2A,B). Similar treatment and analysis of muscle fiber bundles of soleus muscles, however, showed a prominent and significant fragmentation at both investigated age stages (Figures 2A,B). Quantitative analysis of numbers of fragments per NMJ confirmed the non-fragmented status of extensor digitorum longus NMJs (Figures 3A,C). For the NMJs in soleus, it turned out that NMJs with low fragmentation grade (1–5 fragments) are reduced by more than 50% in desmin knock-out mice in comparison with controls (Figures 3B,D). The number of fragmented NMJs was increased by more than 10×-fold in both, NMJs composed of 6–10 and those with more than 10 fragments (Figures 3B,C).

### Desmin Knock-Out Mice Display Enlarged NMJs

**Figure 4:**
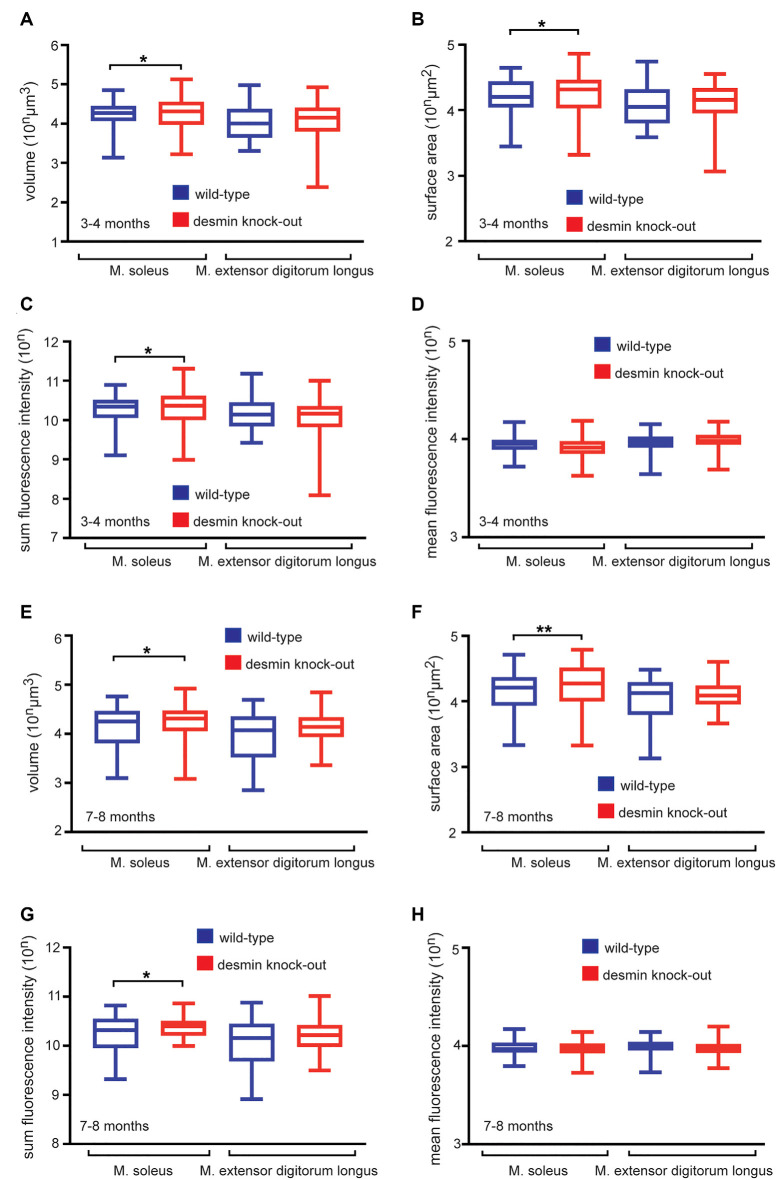
Desmin knock-out mice display enlarged NMJs in soleus muscle. **(A–D)** Hindlimb muscles of 3–4 months old wild-type and homozygous desmin knock-out mice were dissected to stain fiber bundles with BTX. Imaging was done with a fluorescence microscope, and z-stacks were acquired for quantitative 3D morphometry. **(A–D)** Box and Whisker plots depict the recorded average volume, surface, and sum fluorescence intensity per NMJ of indicated genotypes and muscles. Note, surface, volume, and sum fluorescence intensity of NMJs are significantly higher in homozygous desmin knock-out mice in soleus muscle in comparison to wild-type littermates. For extensor digitorum longus muscle a total of 96 wild-types and 89 homozygous NMJs, for soleus muscle 90 wild-type and 103 homozygous NMJs were counted (*n* = 3 mouse pairs). Statistical significance was determined by unpaired two-tailed *t*-test (*P < 0.05). **(E–H)** Hindlimb muscles of 7–8 months old wild-type and homozygous desmin knock-out mice were dissected to stain fiber bundles with BTX. Imaging was done with a fluorescence microscope, and z-stacks were acquired for quantitative 3D morphometry. **(E–H)** Box and Whisker plots depict the recorded average volume, surface, and sum fluorescence intensity per NMJ of indicated genotypes and muscles. Note, surface, volume, and sum fluorescence intensity of NMJs are significantly higher in homozygous desmin knock-out mice in soleus muscle in comparison to the controls. For extensor digitorum longus muscle a total of 102 wild-types and 95 homozygous NMJs, for soleus muscle 99 wild-type and 102 homozygous NMJs were counted (*n* = 3 mouse pairs). Statistical significance was determined by unpaired two-tailed *t*-test (*P < 0.05, **P < 0.01).

To explore the *in vivo* role of desmin for the structural integrity of NMJs, we used the whole-mount BTX stains of desmin knock-out and control mouse muscle fibers for quantitative 3D morphometrical analysis. Notably, the total volume of NMJs appeared significantly enlarged in desmin knock-out mice when compared to controls. This phenomenon was observed in soleus in an age-independent fashion (Figures 4A,E), but not extensor digitorum longus muscles. This change in volume was also expected and reflected by a similar change in their “sum fluorescence intensity” (Figures 4C,G). An increase in surface area would also be expected from larger and at the same time fragmented NMJs, and could be confirmed regardless of age (Figures 4B,F). Further, we asked whether the ablation of desmin protein impaired the intensity of BTX-labeled NMJs, which was analyzed by measuring the “mean fluorescence intensity.” A change of the “mean fluorescence intensity” might reflect a different number of AChRα subunits per NMJ in the absence of desmin. Regardless of the fragmentation status and investigated muscle type, no significant change of the “mean fluorescence intensity” was found in desmin knock-out in comparison with control muscles (Figures 4D,H).

### Gene Set Enrichment Analysis of Transcriptome Data Discloses a Dysregulation of Synaptic Gene Expression

**Figure 5:**
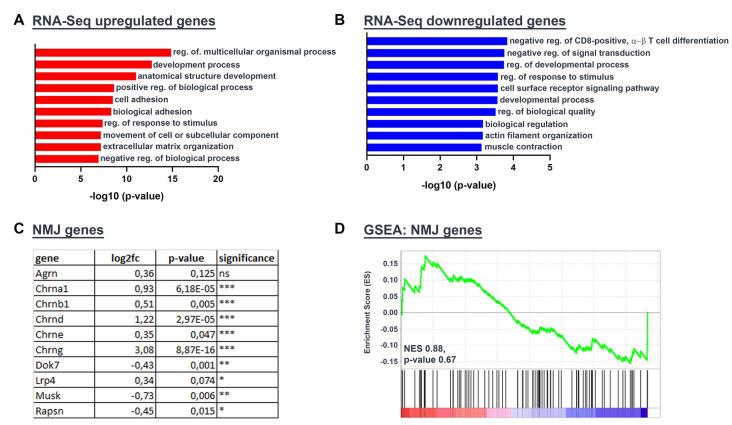
Gene Set Enrichment Analysis of Transcriptome Data discloses a dysregulation of synaptic gene expression. RNA-Seq of five wild-types and five desmin knock-out mouse tissue specimens derived from gastrocnemius/plantaris muscles revealed several differentially regulated genes. **(A,B)** GO Analysis was performed with genes that were up- or downregulated at least 2-fold in desmin knock-out mice (up log2fc ≥1, down log2fc ≤−1). **(C)** The table depicts a set of NMJ-specific genes with their RNA-Seq expression results in the desmin knock-out genotype as compared to wild-type controls [log2fc, *p*-value: *p* (*P ≤ 0.05; **P ≤ 0.01; ***P ≤ 0.001)]. **(D)** Gene set enrichment blot from GSEA analysis of RNA-Seq data with the NMJ gene set. Normalized enrichment score (NES) and *p*-value are listed at the bottom of the blot.

We asked for the reasons for NMJ fragmentation in the desmin knock-out soleus. One of them might be a differentially regulated synaptic gene expression in those muscle fibers. We elucidated the muscle transcriptome of desmin knock-out mice in comparison with the wild-type littermate controls by comparative RNA-Seq experiments. Employing GO term analyses revealed mostly genes belonging to the “regulation of multicellular organismal process” being upregulated and “negative regulation of CD8-positive, alpha-beta T cell differentiation” being downregulated (Figures 5A,B). These topics do not necessarily reflect changes in NMJ biology. Since synaptic gene expression is under-represented in total RNA of muscles due to a very low number of synaptic nuclei in comparison with extrasynaptic nuclei in muscle fibers, a closer look at the synaptic genes and their log2fc was required and indicated that NMJ-specific genes were differentially regulated in the absence of desmin in comparison with controls (Figure 5C). Therefore, Gene Set Enrichment Analysis (GSEA) with an NMJ-specific gene list was carried out (Figure 5D).

Some usual suspects, which control the formation and maintenance of the postsynaptic apparatus at NMJs, are the different subunits of the AChRs, the scaffold protein Rapsyn, the nerve-derived heparan sulfate proteoglycan Agrin, and its receptors, co-receptors, and signaling pathway adaptors, Lrp4, the muscle-specific receptor tyrosine kinase MuSK, and Dok7 (Herrmann et al., 2015). As proof, the desmin mRNA was down-regulated in desmin knock-out muscles in comparison to control; in principle means that it is absent as expected in desmin knock-out mice. Notably, the expression of several synaptic genes, like different Acetylcholine receptor (AChR) subunits is statistically significantly up-regulated in desmin knock-out fibers in comparison with controls (Figure 5C). In contrast, the transcript amounts of the other synaptic candidate genes, like Dok7, Rapsyn, and MuSK, were only slightly down-regulated (Figure 5C).

### Electrophysiological Recordings of Desmin Knock-Out Mice Muscle Fibers Highlight a Higher Decrement After Repetitive Stimulation and Increased Rise Times of mEPPs

**Figure 6:**
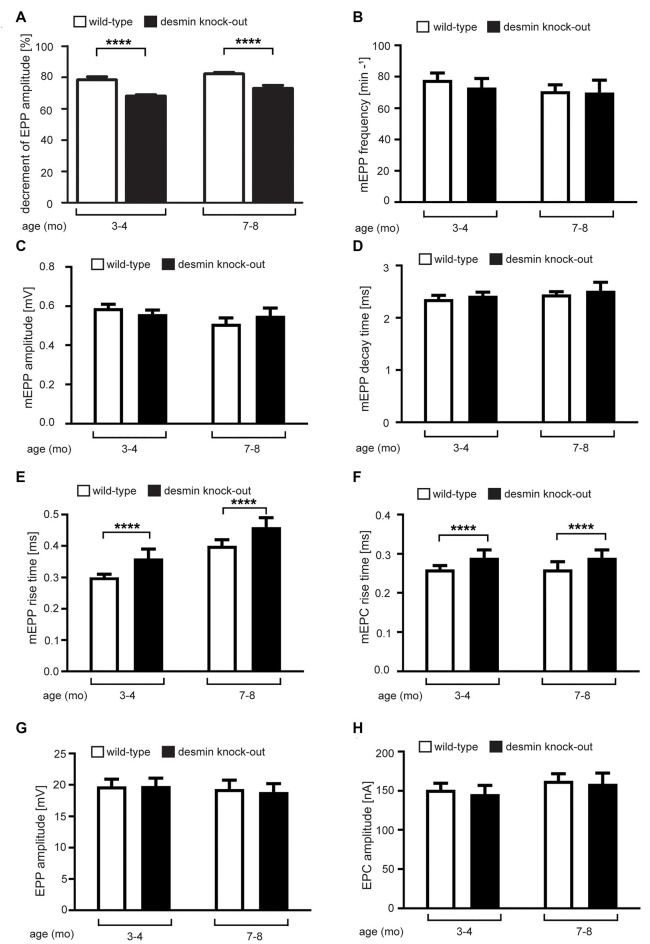
Electrophysiological recordings of desmin knock-out mice muscle fibers reveal a higher decrement after repetitive stimulation and increased rise times of mEPPs. Recordings were done with wild-type and desmin knock-out soleus muscles in 3–4 months-old and 7–8 months-old mice. The graphs show these data as follows: **(A)** Decrement of the endplate potential amplitude, **(B)** mEPP frequency, **(C)** mEPP amplitude, **(D)** mEPP decay time constant, **(E)** mEPP rise time, **(F)** mEPC rise time, **(G)** EPP amplitude, and **(H)** EPC amplitude. Statistical significance was in all cases determined by unpaired two-tailed *t*-test (****P < 0.0001). Note, in desmin knock-out mice the decrement of EPP amplitude was reduced and mEPP/mEPC rise times increased. All other parameters recorded were not changed between wild-type and desmin knock-out genotypes.

To better understand the physiological consequences regarding neuromuscular transmission in desmin knock-out mice, like the potential occurrence of muscle fatigability, we next assessed neuromuscular transmission by repetitive stimulation of the phrenic nerve with 20 Hz trains for 10 s and calculated the decrement of EPP amplitude (Figure 6A). We measured a significant decremental response in both 3–4 and 7–8 months-old desmin knock-out mice in comparison with controls (Figure 6A).

We also asked whether the absence of desmin impinges functional channel characteristics of the AChRs. By monitoring nerve-independently appearing miniature endplate potentials, we neither detected a change in their frequency, amplitude nor decay time constant (Figures 6B–D), but observed a significant increase of the rise times of the miniature EPP (mEPP) in desmin knock-out diaphragms in comparison with controls (Figure 6E). This change was also visible by analyzing the rise time of the mEPC (Figure 6F). Neither input resistance (data not shown) nor EPP or EPC amplitudes were different comparing desmin knock-out with control mice (Figures 6G,H). Quantal content remained also unchanged in the comparison between desmin knock-out and control mice (data not shown).

## Discussion

An extension of our previous work in human patients lacking desmin (Durmus et al., 2016), we here analyzed the structure and function of NMJ in patient-mimicking desmin knock-out mice. For this purpose, two age bins (3–4 and 7–8 month old homozygous desmin knock-out mice) were investigated to elucidate age-dependent effects. In keeping with the myasthenic phenotype in the reported desmin knock-out patients, our analysis depicted NMJ pathology on various levels. Our morphological analysis denoted a fragmentation of NMJs in soleus but not extensor digitorum longus muscle, thus mirroring our previous findings in homozygous desmin R349P knock-in mice, which only express R349P mutant desmin protein (Durmus et al., 2016).

For elucidating structural characteristics of the NMJs in desmin knock-out mice, we applied a 3D quantitative morphology analysis, a methodology which was previously successfully employed (Durmus et al., 2016; Kravic et al., 2016; Giacomazzi et al., 2017; Cescon et al., 2018; Eiber et al., 2019). Here, we found that the increase of NMJ fragmentation not only correlated with an increase of surface area like expected (Figures 4B,F), but there was also an increase of the dimensions of NMJs detectable in desmin knock-out mice in comparison with control mice, reflected by an increase in volume and sum fluorescence intensity (Figures 4A,C,E,G). This observation was also present, though to a lower extent, in the desmin R349P knock-in mice (Durmus et al., 2016).

In this study, we went further and asked for the reason of NMJ fragmentation in the absence of desmin. A possible cause might be different gene expression profiles of synaptic genes. Total RNA sequencing was employed to enlighten the transcriptome profile of desmin knock-out muscles. For technical reasons, the total RNA of the gastrocnemius and plantaris muscles were used for this study. Although it might have been more adequate to use total RNA from soleus, we do not consider that a problem because of the detected changes (see below) and being aware that also the plantaris muscle contains a decent amount of slow fiber types (Huraskin et al., 2016). Almost all AChR subunit genes were upregulated in desmin knock-out mice in comparison to the wild-type littermate controls (Figure 5). This upregulation of AChR subunit genes might indicate a high turnover dynamics of the clustered AChRs, like being reported previously in another context (Eiber et al., 2019). Even the transcription of the AChRγ subunit was up-regulated (Figure 5), like it was shown for R349P desmin knock-in mice, pointing to potential degenerative events ongoing in the absence of desmin. The transcription of other players at NMJs, like MuSK, Dok7, and Rapsyn, was not much affected (Figure 5).

Finally, electrophysiological recordings were used to look for physiological impairments of neuromuscular transmission in desmin knock-out mice, which was not previously studied using desmin R349P knock-in mice (Durmus et al., 2016). For these recordings, soleus muscles of desmin knock-out mice and controls were used. The decrement of endplate potential amplitudes was reduced in desmin knock-out mice in an age-independent manner (Figure 6A), and accompanied by a surprising increase of rising times of mEPPs and mEPCs in desmin knock-out mice (Figures 6E,F). The increased rise times of mEPPs and mEPCs in knock-out mice shown in Figures 6E,F might reflect the observed fragmentation of NMJs, which also accounts for the greater decrement of EPP amplitude with 20 Hz stimulation for 10 s than that in the control. An increase of the rise time might also be the consequence of myofibrillar changes or in a more direct fashion by the specific interaction of desmin with synaptic proteins. We are aware that our data does not clarify all questions. Of course, it is obvious and tempting to assume that only NMJs of the soleus muscle is fragmented, because this muscle is also composed of fast type I fibers compared to the extensor digitorum longus muscle being composed solely of type II fibers. However, our preliminary studies point to an even distribution of fragmented NMJs in the soleus muscle between type I and type II fibers (unpublished data). It remains to understand why then NMJs of soleus are fragmented, but not of extensor digitorum longus muscle. Another question which should be addressed in the future is whether the observed changes between NMJs from soleus and extensor digitorum longus muscle are associated with the changes in the myofiber morphology; in other words, whether the NMJ aberrations could be a result of changes in the fiber structure to understand whether NMJ fragmentation is a primary or secondary effect. To our knowledge, desmin knock-out mice were not analyzed regarding changes in the fiber structure in a muscle-specific manner previously. However, imaging studies did not reveal obvious changes in sarcomere misalignment at the NMJs in flexor digitorum brevis muscles of desmin knock-out mice (Goodall et al., 2012). Our preliminary data (unpublished) do not correlate with severe fiber structure changes being present differently in soleus in comparison to extensor digitorum longus muscle.

In summary, our data highlight the essential role of desmin for the structural and functional integrity of NMJs in man and mice.

## Data Availability Statement

The datasets generated for this study are available by GEO accession number GSE154573 (www.ncbi.nlm.nih.gov/geo/query/acc.cgi?acc=GSE154573).

## Ethics Statement

The animal study was reviewed and approved by Animal protection officer, Sachgebiet Tierschutzangelegenheiten, FAU Erlangen-Nürnberg, AZ: I/39/EE006, TS-07/11.

## Author Contributions

NE, FF, MS, CT, CC, RS, and SH designed and performed the experiments. NE, FF, CC, RS, and SH prepared figures and wrote the original draft. All authors performed review and editing of the manuscript. All authors contributed to the article and approved the submitted version.

## Conflict of Interest

The authors declare that the research was conducted in the absence of any commercial or financial relationships that could be construed as a potential conflict of interest.

## Footnotes

^1^http://www.interfil.org/

^2^http://www.informatics.jax.org/allele/MGI:2159584

^3^www.ncbi.nlm.nih.gov/geo/query/acc.cgi?acc=GSE154573

^4^https://usegalaxy.eu/

^5^http://cbl-gorilla.cs.technion.ac.il/

^6^http://revigo.irb.hr/

^7^https://www.gsea-msigdb.org/gsea/index.jsp

^8^http://amigo.geneontology.org/amigo
